# Supplementary material for: Potential Role of miRNAs in the Acquisition of Chemoresistance in Neuroblastoma
Source: J Pers Med. 2021 Feb 7;11(2):107. doi: 10.3390/jpm11020107 (PMC7916079; doi:10.3390/jpm11020107)
Supplement: Supplementary file 1 [file jpm-11-00107-s001.pdf]

Table S1. miRNAs differently expressed in HTLA-230 and ER-HTLA cells

miRNA	HTLA-230/ ER-HTLA ratio
hsa-miR-26b-5p	47,87
hsa-let-7g-5p	29,21
hsa-let-7f-5p	21,4
hsa-miR-195-5p	19,54
hsa-miR-199a-3p	16,34
hsa-let-7a-5p	16,01
hsa-miR-338-3p	15,99
hsa-miR-126-3p	14,91
hsa-miR-143-3p	14,38
hsa-miR-29b-3p	12,83
hsa-let-7d-5p	12,31
hsa-miR-218-5p	12,3
hsa-let-7i-5p	11,93
hsa-miR-15a-5p	11,38
hsa-miR-199b-5p	10,57
hsa-miR-101-3p	9,879
hsa-miR-126-5p	9,661
hsa-miR-29c-3p	9,267
hsa-miR-30b-5p	9,201
hsa-miR-27b-3p	8,293
hsa-let-7c-5p	8,176
hsa-miR-99a-5p	8,136
hsa-miR-335-5p	8,104
hsa-miR-16-5p	7,894
hsa-miR-19a-3p	7,748
hsa-miR-34c-5p	7,489
hsa-miR-26a-5p	7,442
hsa-miR-374a-5p	7,342
hsa-miR-130a-3p	7,024
hsa-miR-146b-5p	6,954
hsa-miR-29a-3p	6,935
hsa-miR-497-5p	6,924
hsa-miR-5088-5p	0,192
hsa-miR-486-5p	0,133
hsa-miR-6785-5p	0,118
